# RNA-Seq gene expression profiling of HepG2 cells: the influence of experimental factors and comparison with liver tissue

**DOI:** 10.1186/1471-2164-15-1108

**Published:** 2014-12-15

**Authors:** Alexander V Tyakht, Elena N Ilina, Dmitry G Alexeev, Dmitry S Ischenko, Alexey Y Gorbachev, Tatiana A Semashko, Andrei K Larin, Oksana V Selezneva, Elena S Kostryukova, Pavel A Karalkin, Igor V Vakhrushev, Leonid K Kurbatov, Alexander I Archakov, Vadim M Govorun

**Affiliations:** Research Institute of Physico-Chemical Medicine, Malaya Pirogovskaya 1a, Moscow, 119435 Russia; Moscow Institute of Physics and Technology, Institutskii Per. 9, Moscow Region, Dolgoprudny, 141700 Russia; Kazan’ (Volga Region) Federal University, Kremlyovskaya 18, Kazan, 420008 Russia; Orekhovich Institute of Biomedical Chemistry, Pogodinskaya 10, Moscow, 119121 Russia; Shemyakin and Ovchinnikov Institute of Bioorganic Chemistry, GSP-7, Miklukho-Maklaya 16/10, Moscow, 117997 Russia

**Keywords:** HepG2, Liver, Transcriptome, SOLiD, Helicos, RNA-Seq, Differential gene expression, Experimental factors

## Abstract

**Background:**

Human hepatoma HepG2 cells are used as an *in vitro* model of the human liver. High-throughput transcriptomic sequencing is an advanced approach for assessing the functional state of a tissue or cell type. However, the influence of experimental factors, such as the sample preparation method and inter-laboratory variation, on the transcriptomic profile has not been evaluated.

**Results:**

The whole-transcriptome sequencing of HepG2 cells was performed using the SOLiD platform and validated using droplet digital PCR. The gene expression profile was compared to the results obtained with the same sequencing method in another laboratory and using another sample preparation method. We also compared the transcriptomic profile HepG2 cells with that of liver tissue. Comparison of the gene expression profiles between the HepG2 cell line and liver tissue revealed the highest variation, followed by HepG2 cells submitted to two different sample preparation protocols. The lowest variation was observed between HepG2 cells prepared by two different laboratories using the same protocol. The enrichment analysis of the genes that were differentially expressed between HepG2 cells and liver tissue mainly revealed the cancer-associated gene signature of HepG2 cells and the activation of the response to chemical stimuli in the liver tissue. The HepG2 transcriptome obtained with the SOLiD platform was highly correlated with the published transcriptome obtained with the Illumina and Helicos platforms, with moderate correspondence to microarrays.

**Conclusions:**

In the present study, we assessed the influence of experimental factors on the HepG2 transcriptome and identified differences in gene expression between the HepG2 cell line and liver cells. These findings will facilitate robust experimental design in the fields of pharmacology and toxicology. Our results were supported by a comparative analysis with previous HepG2 gene expression studies.

**Electronic supplementary material:**

The online version of this article (doi:10.1186/1471-2164-15-1108) contains supplementary material, which is available to authorized users.

## Background

Human cell lines are widely used to accurately assess the toxic properties and activities of both novel and well-known chemical entities. These experiments are important for the pharmaceutical, chemical, medical and cosmetic industries. Typically, cell line-based assays are preferred to those based on animal models, reflecting the low cost, high reproducibility and bioethical rationale for these assays. One of the most frequently modeled organs of the human organism is the liver, a central organ for the maintenance of chemical homeostasis that participates in the synthesis of proteins and detoxification.

The human hepatoma HepG2 cell line (ATCC HB-8065) has been widely used as an *in vitro* model of the human liver because these cells display a high degree of morphological and functional differentiation *in vitro*, are easy to handle, do not contain viruses and generate reproducible results. This cell line was recovered from a 15-year-old boy with well-differentiated hepatocellular carcinoma. While new promising liver models like pluripotent stem cell-derived hepatocyte-like cells are becoming available [1], HepG2 cells have been used in toxicological studies of liver metabolism and the toxic effects of xenobiotics and in pharmacological studies on drug targeting and carcinogenesis [2].

Gene expression profiling is a powerful tool that has provided numerous insights in previous studies. While RT-PCR and microarrays are commonly used to assess transcriptomic activity, whole-transcriptome high-throughput sequencing (RNA-Seq) provides the most extensive evaluation, with a wide dynamic range and higher sensitivity [3, 4]. Considering the high cost of experiments based on this technology, a thorough experimental design is important. Here, we performed a rigorous assessment of the contribution of each of the main experimental factors *ceteris paribus* to the gene expression profile and compared the results with those reported in existing studies performed using another RNA-Seq platform (Illumina) and previous technology (microarrays). In addition, we derived a characteristic signature for the HepG2 transcriptome compared with liver tissue via differential gene expression analysis, and these results were also compared with previously published results obtained using microarrays.

## Results

### Whole transcriptome of the HepG2 cell line

The 3 technical replicates, HI1, HI2, and HI3, contained 208, 132 and 149 million reads, respectively, with a read length of 50 bp. At the filtering stage, 2.3-4.1% of the total number of reads that aligned with the sequences of human rRNA and SOLiD adapters were discarded. Subsequently, 68%-75% of the reads were successfully mapped to the human genome. More than 17,000 genes were expressed in each sample (18,923 genes when all the replicates were pooled together).

### Validation using ddPCR as a complementary approach

**Figure 1:**
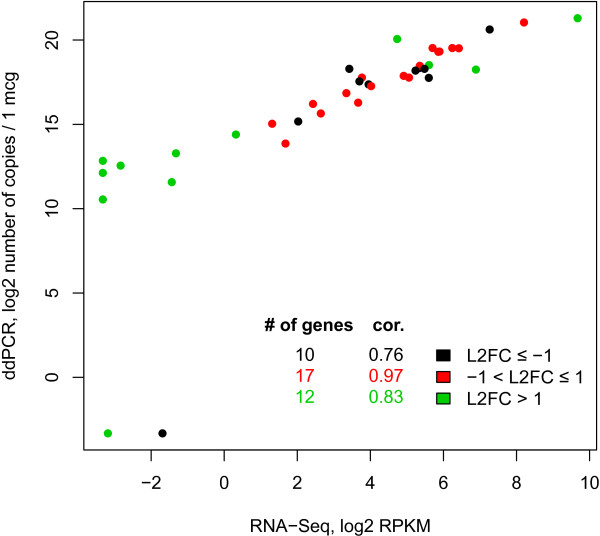
**Comparison of gene expression levels using RNA-Seq.** The RNA-Seq (RPKM values, for HI1 sample) and ddPCR data (number of copies per 1 μg of total RNA) are compared for 45 genes (with pseudocount of 0.1 added, log2). The color of the dots reflects the binning of the respective genes by their log2 fold change (L2FC) in the comparison of HepG2 cell line vs. liver using SOLiD RNA-Seq; for each bin, the number of the genes and Spearman correlation is included. Only the genes identified as differentially expressed are shown.

Droplet digital polymerase chain reaction (ddPCR) is a new technology that facilitates the quantification of target nucleic acids in a sample [5]. For the HepG2 cell line, the ddPCR was implemented to measure 45 selected RNA transcripts. The gene copy number per 1 μg of total RNA varied from 0 to 2,576,000 (Additional file 1: Table S1). A good correlation was observed between the ddPCR and the RNA-Seq three replicates in general (Spearman *r* = 0.943 ± 0.001), as well as when the genes were binned according to their fold change between HepG2 cell line and liver, as detected by SOLiD RNA-Seq, as described below (Figure 1).

### Variation of the transcriptomic profile between the technical replicates and between samples prepared using different methods or different culturing conditions and comparison with liver tissue

For the comparative analysis, we compared the three technical replicates, HI1-3, obtained from a sample cultured at the IBMC with the two RNA-Seq SOLiD datasets generated from a single RNA sample extracted from a HepG2 cell line cultivated at the Research Institute of Physico-Chemical Medicine (RIPCM) in 2011: the first previously unpublished library (id: HR_m, 1 technical replicate) was prepared as described in the Methods section (method M here and below), while the second published library [6] (id: HR_c, 1 technical replicate) was prepared using a different protocol (method C here and below; see Additional file 2: Table S2). In the present study, we also included liver transcriptomes obtained with the SOLiD platform (two technical replicates, ids: L1, L2) [7] using method M. Because the data were obtained on identical platforms using identical formats (SOLiD 4 whole-transcriptome, fragment library, 50 bp reads), we were able to assess the contribution of each of the experimental factor to the quantitative transcriptomic profile. The summary of the SOLiD samples is shown in Table 1. To assess the general variation of gene expression across sequencing platforms, we included two Illumina datasets - one from the ENCODE project [8] (HepG2 cell line, 1 readset, “Transcript Gencode V3c” read counts downloaded from the UCSC Genome browser in GTF format, GEO sample GSM958732) and another from van Delft et al. [9] (HepG2 cell line - control sample, 1 dataset; GEO sample GSM884985).

**Table 1:** **Description of the samples sequenced on the SOLiD 4 platform**

Biological object	Source, date of cultivation and sequencing	[ID of the readset group]: [IDs of readsets]	Sample preparation protocol	Publication and SRA reference
HepG2 cell line	IBMC, 2013	HI: HI1, HI2, HI3	M	previously unpublished, SRR1047450
HepG2 cell line	RIPCM, 2011	HR: HR_m	M	previously unpublished, SRR1254936
HepG2 cell line	RIPCM, 2011	HR: HR_c	C	[6], SRR547993
Liver tissue	IBMC, 2012	L: L1, L2	M	[7], SRR830334

**Figure 2:**
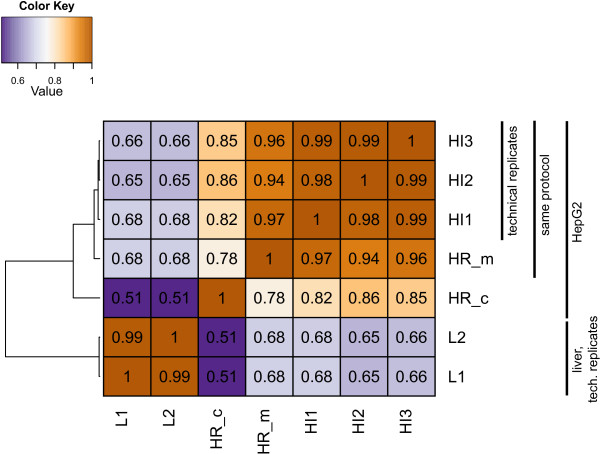
**Dissimilarity heatmap showing the clustering of the transcriptome samples based on the gene expression levels.** Distance metric: 1 - Spearman correlation between the levels of the genes with RPKM > 0.1 in at least one sample; tree constructed using Ward linking; the numbers reflect pairwise correlation values.

All other factors held equal, the lowest Spearman correlation of RPKM values was observed between the HepG2 cell line and liver tissue (group HI + HR_m vs. group L; *r* = 0.67 ± 0.02; Figure 2). The next most dissimilar datasets were derived from HepG2 cells prepared using different protocols (HR_m vs. HR_c; *r* = 0.78), followed by the HepG2 samples from two different laboratories (group HI vs. HR_m; *r* = 0.96 ± 0.01). The technical replicates were the most strongly correlated (within group HI; *r* = 0.99 ± 0.01).

### Comparison of different HepG2 transcriptomes

The effects of experimental conditions and sample preparation were evaluated by the identification of the genes that were differentially expressed across the corresponding pairs of HepG2 datasets. To assess the “laboratory effect”, we compared the HepG2 transcriptomes prepared using the same protocol (method M) originating from laboratories at two different research institutes - IBMC and RIPCM: group HI (the three technical replicates were pooled into one dataset) and HR_m, respectively. Consistent with the general high correlation, most of the expressed genes varied moderately between the datasets, with a log2-fold change of 0.51 ± 0.47 (median ± s.d.; for the 6,947 genes expressed in both datasets with RPKM > 10 in at least one of them). For the 17 housekeeping genes listed in Additional file 3: Table S3, the variation was even lower, showing a log2-fold change of 0.45 ± 0.35 (TUBB gene with zero RPKM values was omitted here; see Additional file 4: Table S4a). The most variable genes (with a fold-change higher than 3) included 70 genes that were up-regulated and 130 genes that were down-regulated in the HI group (Additional file 4: Table S4b) and enriched for small nucleolar RNAs (21% of the differentially expressed genes).

Similarly, to assess the influence of the sample preparation on the gene expression profile, we compared the HepG2 transcriptomes obtained from the same physical sample prepared using two protocols: method M (dataset HR_m) and method C (dataset HR_c). The differences were more significant than between two laboratories (Mann-Whitney’s one-sided test, *P* < 2.2E-16): the log2-fold change for the most highly expressed genes between the datasets was 1.46 ± 2.16 (for the 7,024 genes expressed in both datasets with RPKM > 10 in at least one of them), and the expression of the housekeeping genes was also profoundly changed: the log2-fold change was 0.69 ± 1.29 (similarly, the TUBB gene was also omitted here; Additional file 4: Table S4c). The list of the most variable genes (with fold-changes higher than 3) was more extensive and included 2,374 genes that were up-regulated and 878 genes that were down-regulated in HR_m (Additional file 4: Table S4d). The GO enrichment analysis generated several enriched terms with low adjusted p-values (*adjP* < 0.05); however, the ratio of enrichment, reflecting the effect size, was quite low (*R* < 1.55). Therefore, the underlying biological effect of these differences was not significant.

In order to compare the HepG2 transcriptome obtained using amplification-based platform (SOLiD) with the single-molecule sequencing, we sequenced HepG2 cell line transcriptome on Helicos platform. After calculation of gene expression levels, due to the wide range of order of gene expression level magnitude the genes were binned according to their fold change between HepG2 cell line and liver, as detected by SOLiD RNA-Seq, as described below; the bin-wise correlation was high (0.87 ± 0.12; see Additional file 5: Table S5). Another cross-platform validation was performed by comparing the HepG2 transcriptomes obtained in the present study using SOLiD with the previously published RNA-Seq data obtained from other research groups on an Illumina platform. The HI_1 dataset was selected as a representative of the HI group, and these data were highly correlated with the HepG2 transcriptome from the ENCODE project (Spearman correlation *r* = 0.92, for RPKM vs. FPKM of 18,831 genes resulting after the annotation conversion from RefGene to Ensembl; Additional file 6: Table S6 and Additional file 7: Figure S1). Similarly, HI_1 was highly correlated with the RNA-Seq data from van Delft et al. [9] (*r* = 0.90, for 18,986 genes).

In addition, we compared the RNA-Seq data obtained in the present study with the published In addition, we compared the RNA-Seq data obtained in the present study with the published HepG2 transcriptomes obtained using RNA expression microarrays. A search of the GEO database yielded four studies (GSE5230, GSE6878, GSE6907, GSE9166 – using Affymetrix Human Genome U133 Plus 2.0 Array, Affymetrix Human Genome U133 Plus 2.0 Array, Affymetrix Human HG-Focus Target Array and Affymetrix Human Genome U133A Array, accordingly) containing the microarray gene expression profiles for 14 control HepG2 samples. The correlation of these profiles with the RNA-Seq HepG2 data obtained herein was modest, but it was higher than the correlation with the liver data (0.76 ± 0.04 vs. 0.58 ± 0.03; Additional file 6: Table S6 and Additional file 7: Figure S1). The transcriptomes obtained using method M were slightly more similar to the microarrays than the data obtained using method C (0.77 ± 0.04 vs. 0.73 ± 0.04).

### Hepatocarcinomic signature of HepG2 gene expression profile

Differential gene expression analysis was performed between the liver tissue and HepG2 transcriptomes. The liver group included L1 and L2, while HepG2 was represented by two samples from two laboratories treated as biological replicates: HR_m originated from the RIPCM laboratory, and HI1 - HI3 originated from the IBMC laboratory. The availability of more than one biological replicate of HepG2 in one of the groups allowed the application of the statistical model from the edgeR package. We performed a genewise exact test for differences in the means (with *prior.df* parameter set to 50) to identify the most significant variations. Before the analysis, all respective technical replicates were pooled. Only 15,139 genes covered by >100 reads in at least one sample were considered. With an FDR-adjusted p-value threshold of 0.1, 658 genes were identified as differentially expressed (notably, none of the 18 previously described housekeeping genes were included).

Fifty of these genes were up-regulated in HepG2 cells (Additional file 8: Table S7a). The enrichment analysis of the GO terms using WebGestalt (Additional file 9: Table S8a) yielded 40 significantly enriched biological processes (*adjP* < 0.05). The enriched terms included genes involved in the cell cycle (GO:0007049, *adjP* = 4.10E-07) and cell division (GO:0051301, *adjP* = 1.29E-08). On a more detailed level, the processes related to mitotic cell division were predominant (GO:0000087, *adjP* = 1.91E-11). Particularly, the genes responsible for chromosome condensation, organelle fission and microtubule cytoskeleton organization were overexpressed. The observed phenomena were obviously associated with the active division of HepG2 cells.

From a different angle, the WebGestalt analysis of the associations between the 50 up-regulated genes and diseases showed Aneuploidy among the 10 most enriched disease terms (each of which had *adjP* ≤ 0.005, Additional file 9: Table S8b) (6 genes), Adenoma (6), Chromosome Aberrations (6), Liver Neoplasms (4) and Hepatocellular Carcinoma (4), suggesting an association of these genes with the oncological status of HepG2 cells. To confirm this finding, we characterized the potential associations between the up-regulated genes in HepG2 cells and hepatoma using the liver cancer-related gene signature database, Liverome [10]. For 28 of the 50 genes, increased expression is observed in liver cancer compared with healthy controls, and this increase is associated with the aggressiveness of the tumor and a worse prognosis (this evidence was supported by an average of 4 studies per gene among the 143 publications present in Liverome). These associations from the existing studies also reflected the fact that HepG2 cells are derived from a hepatocarcinoma.

### Response to stimulus is reflected by the gene expression in the liver

The analysis of the 608 genes up-regulated in the liver compared with the HepG2 cell line (Additional file 8: Table S7b) identified 40 enriched GO biological processes (*adjP* < 0.05, Additional file 9: Table S8c). The highest number of the genes (337/608, 55.3%) belonged to the term “response to stimulus” (GO:0050896, *adjP* = 4.16E-21). On a lower level, 192 of these genes were associated with “response to chemical stimulus” (GO:0042221, *adjP* = 4.64E-22), particularly, the 39 genes for “xenobiotic metabolic process” (GO:0009410, *adjP* = 3.19E-21). The activation of the defense response genes was also observed (99 genes are in GO:0006952, *adjP* = 9.13E-17).

Another large enriched term was “small molecule metabolic process” (168 genes in GO:0044281, *adjP* = 5.20E-17), including the 81 genes involved in carboxylic acid metabolism (GO:0019752, *adjP* = 5.55E-15), particularly monocarboxylic acid metabolism (55 genes, GO:0032787, *adjP* = 3.98E-16). Carboxylic acid metabolism is activated in response to the need to provide energy for the detoxification processes and secretory activities of the liver (synthesis and secretion of biologically active substances and blood plasma components), and the cell “boosts” its metabolism to synthesize ATP at an increasing rate. Moreover, carboxylic acids are involved in the biosynthesis of cholesterol, 10% of which is produced in the liver as a precursor for the synthesis of bile acids and steroid hormones.

Consistently, processes associated with electron carrier (25 genes, GO:0009055, *adjP* = 3.16E-09) and oxidoreductase activities (62 genes, GO:0016491, *adjP* = 1.72E-10), primarily provided through the liver cytochrome system, are predominant. The high excretory-secretory activity in the tested liver tissue cells was mirrored by the increased expression of the extracellular region (179 genes, GO:0005576, *adjP* = 2.35E-33) and extracellular matrix (48 genes, GO:0031012, *adjP* = 3.14E-12) genes. Activation of these genes is primarily caused by the expression of the cell surface receptors, the production of blood plasma components, the complement system and interleukins. Furthermore, the enrichment of collagen synthesis (21 genes, GO:0005581, *adjP* = 3.15E-11) is a logical consequence of hepatocyte physiology.

Consistent with the results obtained for the GO terms, the 10 most enriched KEGG pathways (each of which had *adjP* ≤ 1E-11) included “Drug metabolism - cytochrome P450”, “Metabolism of xenobiotics by cytochrome P450” and “Drug metabolism - other enzymes” (Additional file 9: Table S8d). Moreover, the increased expression of the genes involved in the “Retinol metabolism” pathway is consistent with the breakdown of dietary vitamin A (all-trans-retinol) in the small intestine and the release of retinol. Retinol is transported to the liver in the form of chylomicrons where it is converted into retinyl esters that are accumulated in liver cells [11].

Among other KEGG pathways, the observed enrichment of the “Steroid hormone biosynthesis” pathway primarily reflects the presence of enzymes responsible for steroid and bilirubin inactivation, another physiological function of the liver. The cholesterol synthesis enzymes were also up-regulated. The up-regulation of the “Complement and coagulation cascades” pathway also reflects the physiological function of the liver, involving the synthesis and secretion (excretion) of essential plasma proteins, including the complement system and clotting factors.

## Discussion

Transcriptomic profiling based on high-throughput sequencing (RNA-Seq) facilitates the analysis of gene expression at an unprecedented high dynamic range. Global initiatives, including ENCODE, have provided an extensive inventory of human tissues and helped to identify differentially expressed genes with subsequent applicability in clinical practice. We applied RNA-Seq using a SOLiD 4 platform for deep transcriptomic profiling of HepG2 cells, a widely used laboratory cell line. A large amount of transcriptomic data for HepG2 cells is available, and most of these data have been generated using RNA hybridization microarrays. Recently, HepG2 transcriptomes have been examined using high-throughput sequencing on the Illumina [8, 9] and SOLiD [6, 7] platforms. The experiments described herein were performed on a single platform (SOLiD 4) using the same sequence format to eliminate the choice of platform as a confounding factor and focus on the influence of the other experimental factors for the evaluation of the HepG2 transcriptome and the determination of transcriptomic differences between the liver and HepG2 cells as a model system for this organ.

A comparison of the transcriptomic data obtained using the same sequencing platform revealed that the sample and sequencing library preparation protocol, not the “laboratory effect” as expected, had the highest contribution to the HepG2 gene signature. The minor differences in the expression profiles between the laboratories (HI vs. HR_m) likely reflect fluctuations in HepG2 cell metabolism between the two cultivation rounds and the RNA extraction. Given the high fraction of snoRNAs among the differentially expressed genes, the fact that differential RNA-Seq analysis is inherently biased against profiling of short genes [12] could also contribute to these differences; however, the RPKM values for these snoRNA genes were not outlying (Additional file 10: Figure S2). In contrast, the differences between the preparation methods (HR_m vs. HR_c) were much larger than those between the laboratories: 3,252 differentially expressed genes were identified. However, the analysis of this group of genes did not reveal any biological relevance, consistent with the idea that the source of the variation was primarily technical and associated with the differences in the protocols’ steps (see Additional file 2: Table S2). Notably, the workflow of sample preparation method C is similar to the method used for microarrays, potentially accounting for the lower correlation between RNA-Seq using method M and microarrays than between method C and microarrays. Our analysis was limited to examining two protocols in one laboratory and one of the protocols in another laboratory. Obviously, a comparison of a greater number of protocols in each of a greater number of laboratories is needed to clarify the observed effects of these experimental factors. Without denying the existence of protocols that do produce highly similar results, we would like to emphasize that the significant differences observed between the two established methods point out the need for a careful planning of sample preparation in RNA-Seq experiments.

The comparison of the HepG2 transcriptome obtained using the SOLiD platform with the results of two published studies based on the Illumina platform [8, 9] showed high correlation (*r* > 0.9), suggesting the consistency and comparability of the RNA-Seq data generated using these two prevalent high-throughput platforms, despite the effect of possible differences in biological material and sample preparation protocols. Importantly, the correlation with Helicos sequencing results was also high, supporting the comparability of the data obtained using amplification-based and single-molecule sequencing approached. The lower correlation with the microarray data was consistent with the published differences between the two technologies for expression profiling [9].

An omics-based comparison of the liver tissue (hepatocytes) with traditional cell models is crucial for assessing the potential limitations of the *in vitro* experiments. Here, we compared the HepG2 transcriptomic signature with that obtained with liver tissue biopsy material [7]. The majority of the genes that were up-regulated in HepG2 cells were associated with cancer, while the genes that were up-regulated in the liver were associated with xenobiotic metabolism. Considering these large groups, researchers should be wary when designing transcriptomic experiments using HepG2 cells as a liver model. If the target genes are associated with the oncology of interest, then the expression levels of these genes will be a strongly biased estimate of their levels in the liver. This fact has important implications for drug, toxicity and carcinogenicity testing. In addition, the increased levels of the genes associated with xenobiotic inactivation in the liver transcriptome could reflect the fact that the samples were biopsies obtained from the patients who were likely to receive medical treatment.

We compared the list of the genes that were differentially expressed between HepG2 cells and the liver (SOLiD RNA-Seq) to the corresponding results obtained in another study of HepG2 cells and hepatocytes using microarrays [13]. The analysis revealed that 27 of the 50 genes that were up-regulated and 271 of the 608 genes that were down-regulated in HepG2, as assessed using the RNA-Seq, were also detected using microarrays (among the 3,559 up-regulated and 2,540 down-regulated genes, respectively). None of the up-regulated genes identified in RNA-Seq was among the down-regulated genes identified through microarray, and only 2 of the down-regulated genes were among the up-regulated genes detected by microarray. While the RNA-Seq analysis was more specific, and the number of differentially expressed genes identified in the RNA-Seq analysis was an order of magnitude lower, the significance testing confirmed the general consistency of the results from the two studies (Pearson’s chi-squared test with simulated p-value, chi-squared = 1183.63, *p* = 5E-4; see Methods for details).

A majority of the 23 genes that were up-regulated in HepG2 cells, identified through RNA-Seq but not microarray analysis, encoded proteins that are associated with active mitotic cell division, particularly the M phase of the mitotic cell cycle. This finding is logically explained by the biological difference between the samples compared in the two studies: in the present study, we compared a growing HepG2 cell line with a stationary liver biopsy sample, while Costantini et al. [13] compared two cell lines, each dividing at a different rate; thus, these genes were not differentially expressed.

In contrast, among the 391 genes that were down-regulated in HepG2 cells, identified through RNA-Seq but not microarray analysis, the largest group (181/391, 46.3%) was associated with the GO category “response to any stimulus”, with a majority of the genes responsible for detoxification. The next largest group comprised 71 genes (18.2%) belonging to the GO term “immune system process”. A high proportion of these genes are involved in the synthesis of blood plasma proteins and components of the immune system. Both of these processes are physiological functions of the liver under normal conditions. Therefore, the reduced expression of these genes in HepG2 cells compared with liver tissue is logical. The conditions for hepatocyte cultivation *in vitro* might have led to inactivation of these metabolic pathways, thus decreasing the differences in the HepG2 cell line.

In addition to the choice of transcriptome profiling technology and differences in the biological objects, other factors likely contribute to the variations between the two studies. One factor is the availability of biological replicates: Costantini et al. [13] included 4 replicates per group, but these replicates were technical, not biological. In contrast, in the present RNA-Seq study, we had 1 sample for liver and 2 biological replicates for HepG2 cells. The availability of these biological replicates, i.e., two HepG2 samples from two different laboratories, allowed a more specific detection of the HepG2 gene expression signature based on the natural variability of the biological object. This fact must have contributed to the identification of a lower number of differentially expressed genes in the present study.

Secondly, the statistical models were quite different between the studies. Specifically, Costantini et al. [13] used an “Illumina Custom” error model, which considers the gene levels to be distributed normally across the biological replicates; the details about the multiple testing adjustment were not specified. In the present study, we used edgeR software, which accounts for overdispersion across biological replicates, with application of multiple testing correction to the resulting gene list (FDR method). This approach implies a higher specificity of the resulting set of differentially expressed genes.

## Conclusions

In the present study, we demonstrated that the transcriptomic profiles obtained using RNA-Seq are generally invariant across the sequencing platforms (SOLiD and Illumina) and are reproducible for HepG2 cell lines across different laboratories. An evaluation of the experimental factors showed that the sample preparation protocol is the strongest factor influencing the gene expression profile.

We identified the characteristic differences in gene expression between HepG2 cells and liver tissue. Although most genes showed insignificant changes, the genes showing significantly different expression outline the cancer-associated signature that should be highly considered when choosing the application of HepG2 cells as a model system for liver transcriptomics.

Comparisons with other studies showed that the SOLiD sequencing data are consistent with previous results and can be used in future studies as an external control for estimating biological variability and the quality of new data to optimize experimental design, including the calculation of coverage depth and number of replicates.

## Methods

### Cell culture

The human hepatocellular carcinoma HepG2 cell line was obtained from the collection of the Orekhovich Institute of Biomedical Chemistry (IBMC). Frozen cells were thawed and expanded in DMEM/F12 supplemented with 10% fetal bovine serum (FBS) and 100 units/ml penicillin/streptomycin (all from Gibco, Carlsbad, CA) in a humidified CO_2_ incubator under standard conditions (5% CO_2_, 37°C).

### RNA isolation and analysis

Total RNA was isolated from the HepG2 cells using the RNeasy mini kit (Qiagen GMBH, Hilden, Germany) according to the manufacturer’s instructions, yielding 150 μg of total RNA. The amount of total RNA was determined using a NanoDrop 1000 spectrophotometer (NanoDrop, Wilmington, DE). The quality of the RNA preparation was evaluated using the Agilent RNA 6000 Nano Kit on an Agilent Bioanalyzer 2100 system (Agilent, Palo Alto, CA). The quality of the preparation was considered to be high based on an RIN value of 8.7.

### mRNA enrichment

mRNA was enriched from total RNA using the MicroPoly(A)Purist Kit (Ambion by Life Technologies, Houston, TX, USA) in triplicate according to the manufacturer’s instructions. Two rounds of enrichment were performed. The RNA concentration was measured using a Qubit 2.0 Fluorometer (Life Technologies, Carlsbad, CA) with the Quant-iT RNA Assay Kit, 5–100 ng. The RNA quality and rRNA depletion were estimated using the 2100 Bioanalyzer system (Agilent Technologies) and the RNA 6000 Pico kit. A total of 1.1, 1.4 and 1.4 μg of mRNA was obtained from 150 μg total RNA.

### Library preparation and RNA sequencing

The mRNA fragment library was prepared using the SOLiD Total RNA-Seq Kit (Ambion) according to the manufacturer’s instructions. The mRNA fragment library was sequenced on a SOLiD 4 platform (Life Technologies) according to the manufacturer’s instructions, with three technical replicates (sample identifiers: HI1, HI2, HI3; group identifier: HI). The length of each read was 50 bp.

### Droplet digital PCR (ddPCR)

For cDNA preparation and ddPCR, the RNA was treated with DNase I (Thermo Fisher Scientific, Waltham, MA) and subsequently used for cDNA synthesis with H-minus Mu-MLV reverse transcriptase (Thermo Fisher Scientific) and random hexanucleotides according to the manufacturer’s instructions. The ddPCR reaction mixtures were prepared in 20-μl volumes containing 1X ddPCR Supermix (Bio-Rad Laboratories, Hercules, CA), 0.3 μM of each primer and probe (Additional file 1: Table S1), and 0.6-1 μg of cDNA. The droplet generation and droplet reading for ddPCR were performed according to the manufacturer’s instructions using Bio-Rad reagents.

### Read mapping and gene expression analysis

The color-space reads were mapped to the human genome (version hg18) using Lifescope software (Life Technologies) with RefSeq genome annotation obtained from the UCSC browser, yielding both raw read counts and RPKM values for each of the 24,628 genes. The statistical analysis was performed in R [14]. Differential gene expression was analyzed using two methods. When at least two biological replicates were available for at least one comparison group (i.e., analysis between the liver and cell line samples), the analysis was performed using the edgeR package [15] based on read counts (multiple comparison adjustment correction was performed using the false discovery rate [FDR]; the difference was considered significant if the adjusted p-value did not exceed 0.05). In other cases, the log of the RPKM fold-change for each gene between the two samples was assessed for the major genes (genes expressed in both samples, with an RPKM higher than a threshold value in at least one sample). The gene set enrichment analysis was performed using WebGestalt [16], with the Entrez Gene set as a reference, using a hypergeometric test with Benjamini-Hochberg multiple comparison adjustment correction (significance criterion: adjusted p-value *adjP* < 0.05) and the maximum number of significant hits *M* = 40.

### Comparison with the published data

For comparison of the Illumina datasets with the SOLiD gene expressions profiles, the EnsEMBL gene names available for the ENCODE (GENCODE annotation) and van Delft et al. (EnsEMBL v58 genome) data were converted to HGNC gene names using biomaRt R package. For comparison of the four microarray datasets downloaded from GEO with the SOLiD gene expressions profiles, the microarray gene expression levels were averaged across the probes and assigned HGNC gene names using GEOquery R package.

To check the general consistency of the differential expression analysis results obtained in our study using RNA-Seq and in the study of Costantini et al. [13] using microarrays, we considered the direction of change (if detected) for each of the genes that were included in the RefSeq annotation for RNA-Seq (n = 24,628). For RNA-Seq, the genes up-regulated in HepG2 cell line in comparison with the liver were assigned tag “1” and the down-regulated genes “-1”; all the other genes were assigned “not significant” tag. The same procedure was performed for microarrays (after the conversion of the gene names from microarrays that were not found in the RefSeq annotation using org.Hs.eg.db R package). The resulting two vectors of length *l* = 24,628 were transformed into 3 × 3 contingency table and tested for independence using Pearson’s chi-squared test with simulated p-value (based on 2,000 replicates).

### Availability of supporting data

The SOLiD sequence data (HI and HR_m) are available in the Sequence Read Archive: SRA accession numbers SRR1047450 and SRR1254936, respectively. The Helicos sequence data are available at http://download.ripcm.com/HepG2_article/.

## Electronic supplementary material

Additional file 1: Table S1: ddPCR gene copy number. (XLS 34 KB)

Additional file 2: Table S2: Step-by-step comparison of the two sample preparation protocols used for SOLiD RNA-Seq. (DOC 39 KB)

Additional file 3: Table S3: List of the housekeeping genes. (XLS 8 KB)

Additional file 4: Table S4: Change of gene expression associated with various factors. a. List of the housekeeping genes for the datasets from two laboratories (HI and HR_m), with the RPKM and log2 fold change. b. List of the most variable genes between the datasets from two laboratories (HI and HR_m), with the RPKM and absolute value of log2 fold change. Table S4c. List of the housekeeping genes for the datasets with two sample preparation methods (HR_c and HR_m), with the RPKM and log2 fold change. Table S4d. List of the most variable genes between the datasets obtained with two sample preparation methods (HR_c and HR_m), with the RPKM and absolute value of log2. (XLS 332 KB)

Additional file 5: Table S5: Spearman correlation of HepG2 gene expression levels between SOLiD and Helicos. (XLS 28 KB)

Additional file 6: Table S6: Spearman correlation between the SOLiD gene expression profiles and the published Illumina RNA-Seq and microarray datasets. (XLS 266 KB)

Additional file 7: Figure S1: Hierarchical dendrogram of Spearman correlation between SOLiD, Illumina and microarray gene expression levels. (PDF 211 KB)

Additional file 8: Table S7: Genes differentially expressed in cultured HepG2 cells in comparison to the liver. a. List of genes that were upregulated in cultured HepG2 cells in comparison to the liver (including the log2-fold-change in liver relative to HepG2, the average log2-counts-per-million, the two-sided p-value and its adjusted version, with cutoff FDR > 0.1). b. List of genes that were upregulated in the liver in comparison to the cultured HepG2 cells (including the log2-fold-change in liver relative to HepG2, the average log2-counts-per-million, the two-sided p-value and its adjusted version, with cutoff FDR > 0.1). (XLS 102 KB)

Additional file 9: Table S8: Enrichment analysis of differentially expressed genes. a. Gene Ontology (GO) terms enriched in HepG2 cells in comparison to the liver according to WebGestalt analysis. b. Disease-associated gene sets enriched in HepG2 cells in comparison to the liver according to WebGestalt analysis. Table S8c. Gene Ontology (GO) terms enriched in the liver in comparison to HepG2 cells according to WebGestalt analysis. Table S8d. KEGG pathways enriched in the liver in comparison to HepG2 cells according to WebGestalt analysis. (XLS 878 KB)

Additional file 10: Figure S2: Plot of gene length vs. RPKM value for HI1 and HR_m samples. (PDF 306 KB)

## Abbreviations

RNA-Seq: RNA sequencing
RT-PCR: Real-time polymerase chain reaction
ddPCR: Droplet digital PCR
RPKM: Reads per kilobase model per million mapped reads
FDR: False discovery rate
KEGG: Kyoto Encyclopedia of Genes and Genomes
SRA: Sequence Read Archive
ENCODE: The Encyclopedia of DNA Elements
GTF: General Transfer Format
GEO: Gene Expression Omnibus
GO: Gene Ontology.
