# Supplementary material for: RNA-Seq gene expression profiling of HepG2 cells: the influence of experimental factors and comparison with liver tissue
Source: BMC Genomics. 2014 Dec 15;15(1):1108. doi: 10.1186/1471-2164-15-1108 (PMC4378340; doi:10.1186/1471-2164-15-1108)
Supplement: Supplementary file 2 — Additional file 2: Table S2: Step-by-step comparison of the two sample preparation protocols used for SOLiD RNA-Seq. (DOC 39 KB) [file 12864_2014_6832_MOESM2_ESM.doc]

| Table S2. Step-by-step comparison of the two sample preparation protocols used for SOLiD RNA-Seq. | | |
| --- | --- | --- |
| # | Method M | Method C |
| 1 | Total RNA extraction. Total RNA was isolated from the HepG2 cells using the RNeasy mini kit (Qiagen) according to the manufacturer’s instructions, yielding 150 μg of total RNA. The amount of total RNA was determined using a NanoDrop 1000 spectrophotometer (NanoDrop). The quality of the RNA preparation was evaluated using the Agilent RNA 6000 Nano Kit on an Agilent Bioanalyzer 2100 system (Agilent). | Total RNA extraction. Total RNA was isolated from the HepG2 cells using the RNeasy mini kit (Qiagen) according to the manufacturer’s instructions, yielding 150 μg of total RNA. The amount of total RNA was determined using a NanoDrop 1000 spectrophotometer (NanoDrop). The quality of the RNA preparation was evaluated using the Agilent RNA 6000 Nano Kit on an Agilent Bioanalyzer 2100 system (Agilent). |
| 2 | mRNA enrichment. mRNA was enriched from total RNA using the MicroPoly(A)Purist Kit (Ambion) according to the manufacturer’s instructions. Two rounds of enrichment were performed. The RNA concentration was measured using a Qubit 2.0 Fluorometer (Life Technologies) with the Quant-iT RNA Assay Kit, 5–100 ng. The RNA quality and rRNA depletion were estimated using the 2100 Bioanalyzer system (Agilent Technologies) and the RNA 6000 Pico kit. | cDNA preparation. Double-stranded amplified cDNA was obtained using Mint-2 Kit (Evrogen, Moscow, Russia) according to the manufacturer’s instructions. The DNA concentration was measured using a Qubit 2.0 Fluorometer (Life Technologies) with the Quant-iT dsDNA BR Assay Kit with a range of 2-1000 ng. |
| 3 | Library preparation. The mRNA fragment library was prepared using the SOLiD Total RNA-Seq Kit (Ambion) according to the manufacturer’s instructions. The procedure included the following steps:  - mRNA fragmentation;  - specific adapters ligation;  - preparation of double-stranded cDNA using universal primers;  - initial library amplification. | Library preparation. The cDNA fragment library was prepared using the SOLiD TM Fragment Library Construction Kit and SOLiD Fragment Library Oligos Kit (Life Technologies) according to the manufacturer’s instructions. The procedure included the following steps:  - double-stranded cDNA fragmentation;  - size selection;  - specific adapters ligation;  - initial library amplification. |
