# Supplementary figures and images for: RNA-Seq gene expression profiling of HepG2 cells: the influence of experimental factors and comparison with liver tissue
Source: BMC Genomics. 2014 Dec 15;15(1):1108. doi: 10.1186/1471-2164-15-1108 (PMC4378340; doi:10.1186/1471-2164-15-1108)

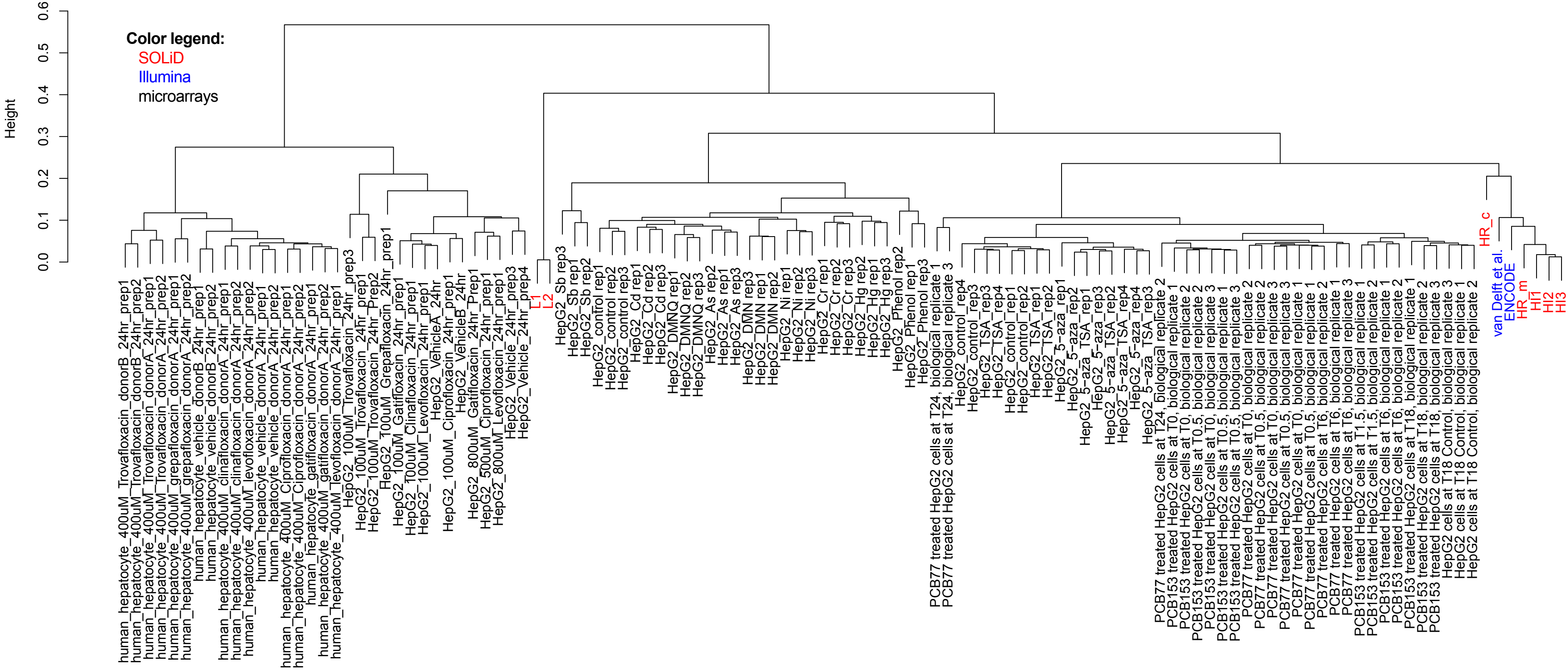

Supplement: Supplementary file 7 — Additional file 7: Figure S1: Hierarchical dendrogram of Spearman correlation between SOLiD, Illumina and microarray gene expression levels. (PDF 211 KB) [file 12864_2014_6832_MOESM7_ESM.pdf]

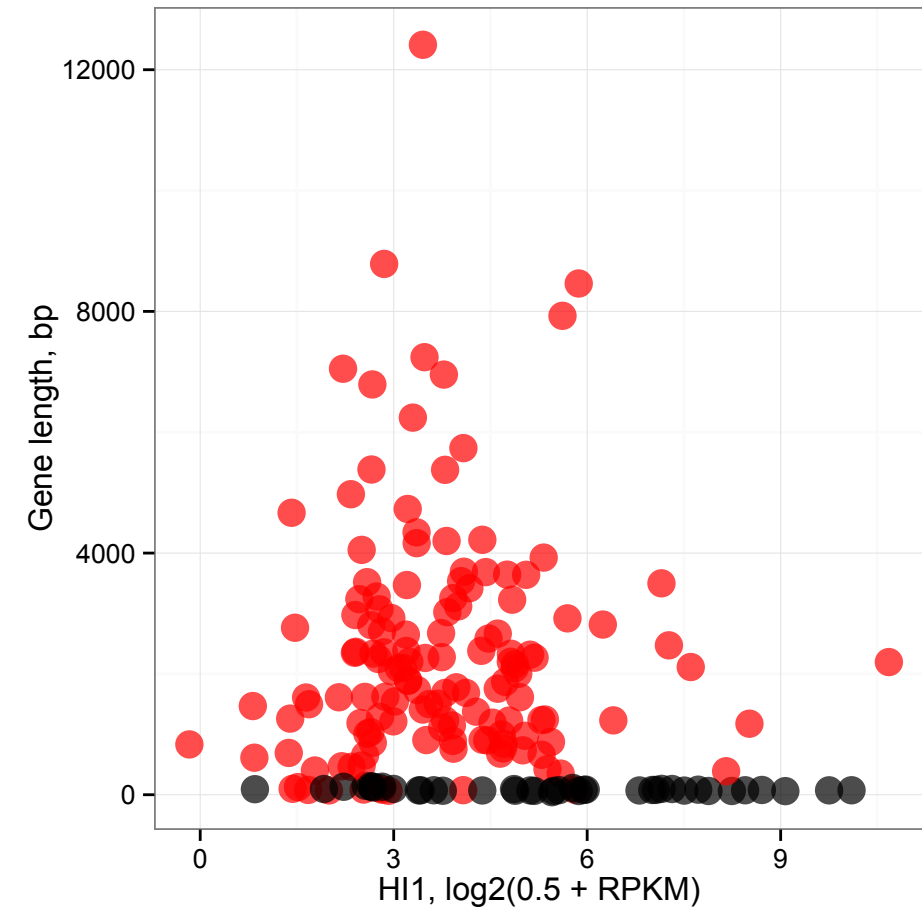

**Color legend**  
● SNOR\*  
● other genes

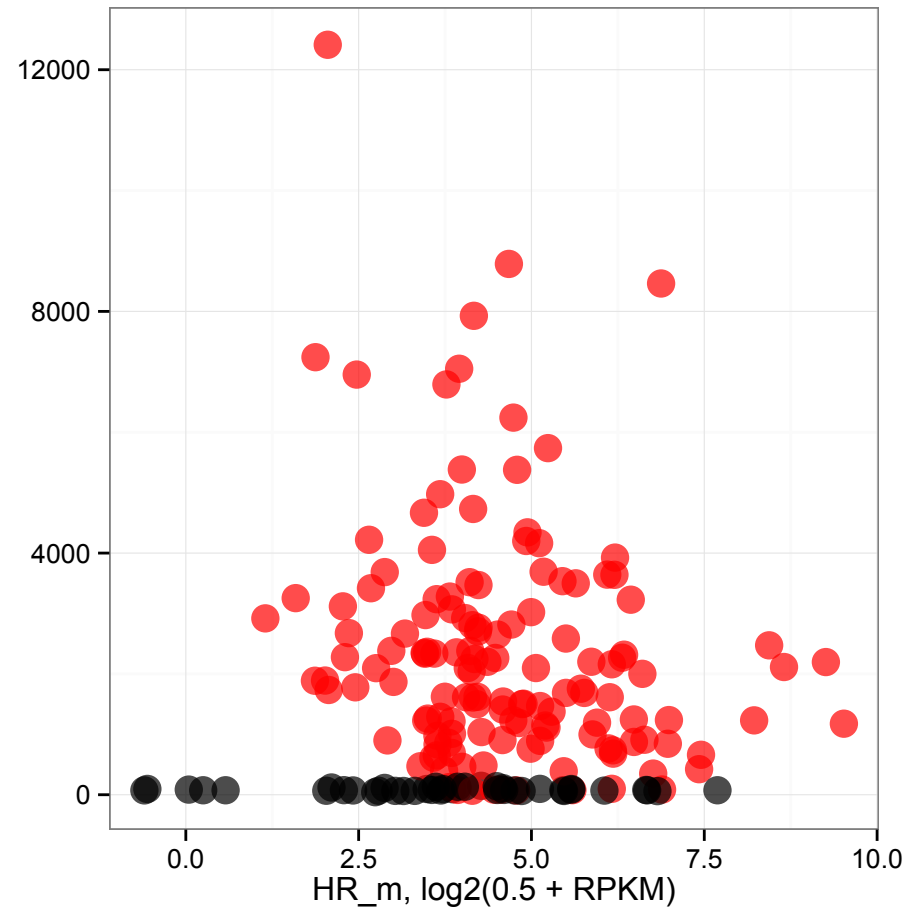

Supplement: Supplementary file 10 — Additional file 10: Figure S2: Plot of gene length vs. RPKM value for HI1 and HR_m samples. (PDF 306 KB) [file 12864_2014_6832_MOESM10_ESM.pdf]
